# Supplementary material for: Development of rechargeable high-energy hybrid zinc-iodine aqueous batteries exploiting reversible chlorine-based redox reaction
Source: Nat Commun. 2023 Apr 3;14:1856. doi: 10.1038/s41467-023-37565-y (PMC10070632; doi:10.1038/s41467-023-37565-y)
Supplement: Supplementary file 1 — Supplementary Information [file 41467_2023_37565_MOESM1_ESM.pdf]

Supplementary Information

Development of rechargeable high-energy hybrid zinc-iodine aqueous batteries exploiting reversible chlorine-based redox reaction

Guojin Liang[1], Bochun Liang[1], Ao Chen[1], Jiaxiong Zhu[1], Qing Li[1], Zhaodong Huang[1], Xinliang Li[1], Ying Wang[2]*, Xiaoqi Wang[3], Bo Xiong[3], Xu Jin[3], Shengchi Bai[3], Jun Fan[1]*, Chunyi Zhi[1,4]*

[1]Department of Materials Science and Engineering, City University of Hong Kong, 83 Tat Chee Avenue, Kowloon, China

[2]State Key Laboratory of Rare Earth Utilization, Changchun Institute of Applied Chemistry, Chinese Academy of Sciences, Changchun 130022, China

[3]Research Institute of Petroleum Exploration & Development (RIPED), Research Center of New Energy, No. 20 Xueyuan Road Haidian District, Beijing, 100083, P. R. China

[4]Center for Advanced Nuclear Safety and Sustainable Development, City University of Hong Kong, Kowloon, Hong Kong, 999077, China

*email: ywang_2012@ciac.ac.cn; junfan@cityu.edu.hk; cy.zhi@cityu.edu.hk

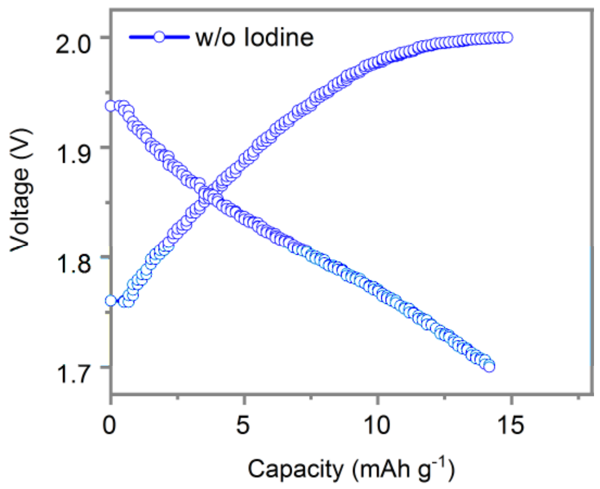

Supplementary Figure 1. GCD profile of the two-electrode pouch Zn||AC cells in 30 m $ZnCl_2$ at 0.5 A g_{AC}^{-1} cycled and 25 °C, where the positive electrode was bare I-absent AC coated on Ti-mesh. The specific current and speific capcity (14.6 mAh g^{-1}) was based on the mass of AC, which exhibited no discharging plateau, and the Cl redox reactions may slightly contribute to the capacity of the bare AC electrode.

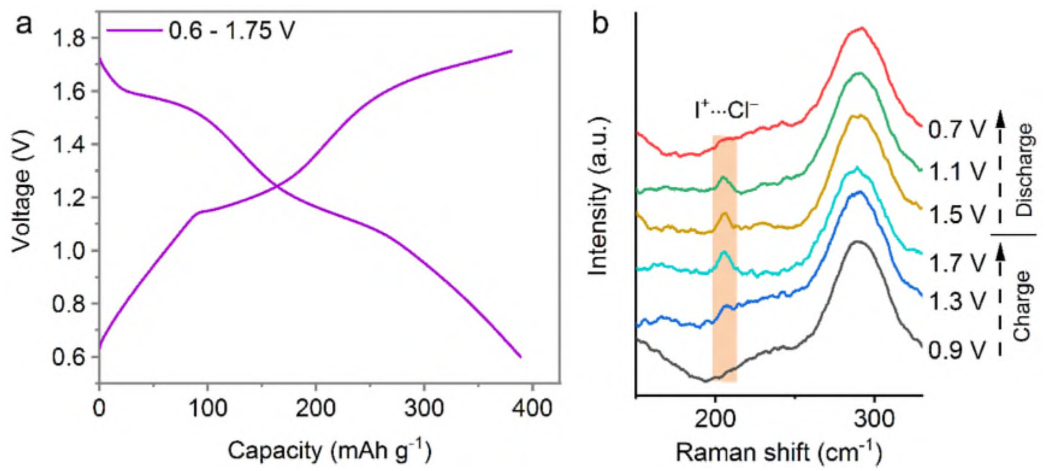

Supplementary Figure 2. (a) GCD profile of the two-electrode pouch Zn||Cl-I cells, which was cycled in 30 m $ZnCl_2$ within the potential range from 0.6 to 1.75 V at 0.5 A g_{I2}^{-1} and 25 °C. The specific current and speific capcity was based on the mass of iodine. (b) In situ Raman characterization to trace the corresponding GCD curves. The peak at 204 cm^{-1} is assigned to the presence of $I^{+}Cl^{-}$ species, which emerged during charging and then vanished during discharging.

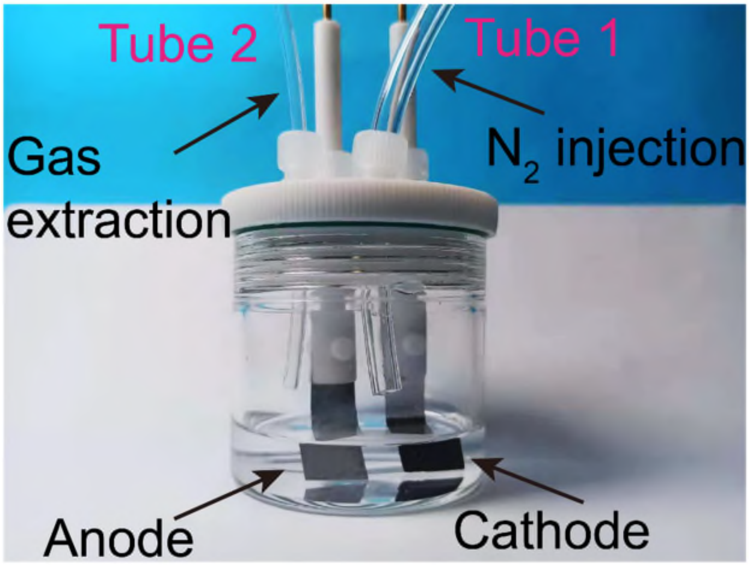

Supplementary Figure 3. Online electrolytic cell with flooded electrolyte is applied to detect the presence of Cl_2 evolution of I-containing and I-absent cathodes respectively, and the anode is Zn foil, while the cell is placed in the oven under 50 °C. Two tubes are applied to conduct the gas loop that the Tube 1 is applied for N_2 injection and Tube 2 for gas extraction.

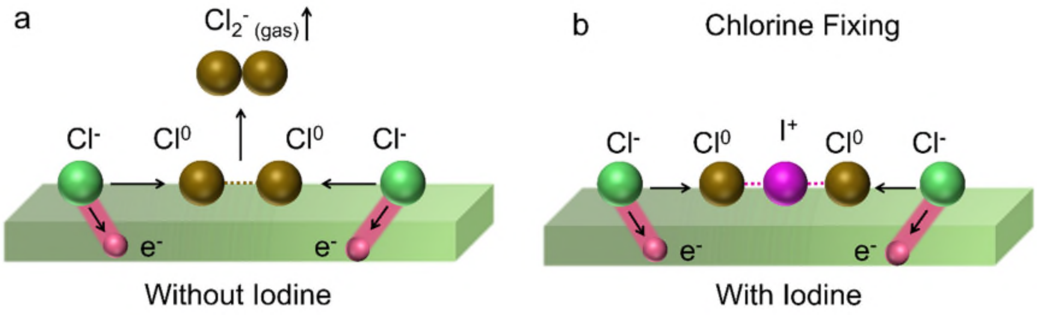

Supplementary Figure 4. Schematic illustration of the Cl^0 evolving to gaseous Cl_2 molecules (a), and being fixed by I^+ (b). Here, the valence state of I is +1 due to the working voltage range is 1.7 to 2 V to preserve the I^+ as shown in Supplementary Figure 2 and Figure 3a. The green substrate in the figure is the surface of activated carbon.

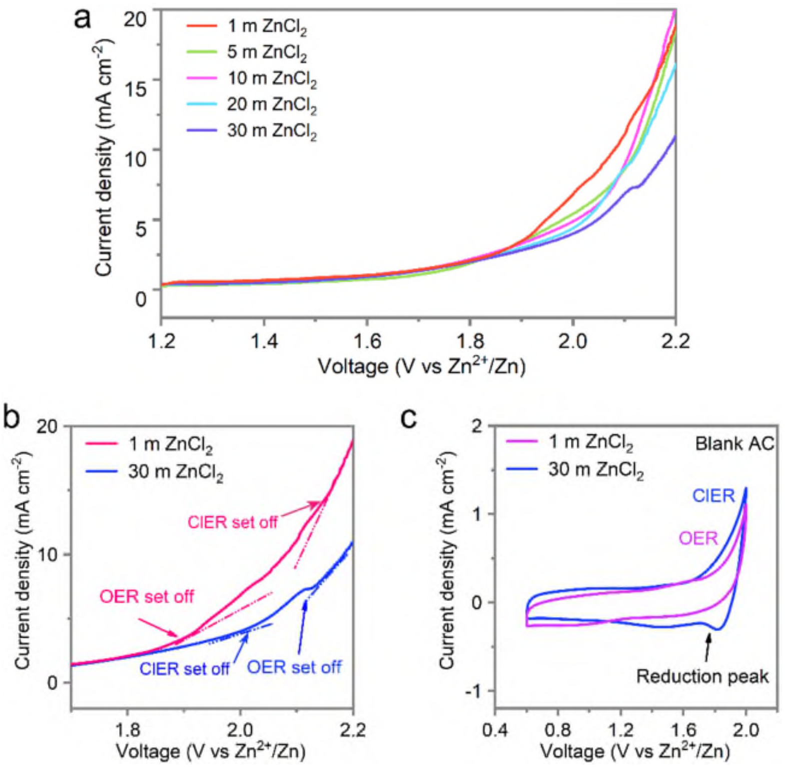

Supplementary Figure 5. (a) LSV profiles in different concentration electrolytes to detect the OER and Cl oxidation reactions at 0.5 mV s^{-1} and 25 °C. It was tested in a two-electrode Zn||Ti cells pouch cell configuration by coupling Ti foil with Zn. (b) The zoom-in voltage region of (a) to analyze the set-off potential of Cl oxidation reactions in dilute (1 M) and concentrated (30 m) electrolytes. The set-off potential of OER in 1 M $ZnCl_2$ electrolyte is determined by the pronounced change of the slope at around 1.95 V, indicating the current density origination has changed from capacitive adsorption to redox reaction. In 30 m $ZnCl_2$ electrolyte, OER is suppressed with the set-off potential located at around 2.1 V due to the small but noticeable peak after the Cl oxidation reaction (arrowed out). On the other side, the ClER decreased from 2.13 V of the 1 M $ZnCl_2$ electrolyte to 1.98 V of the 30 m $ZnCl_2$ electrolytes. (c) CV curves of the blank AC electrode in two different electrolytes in the two-electrode pouch Zn||AC cells, where one cathodic peak exists in 30 m electrolyte and is absent in the 1 M electrolyte.

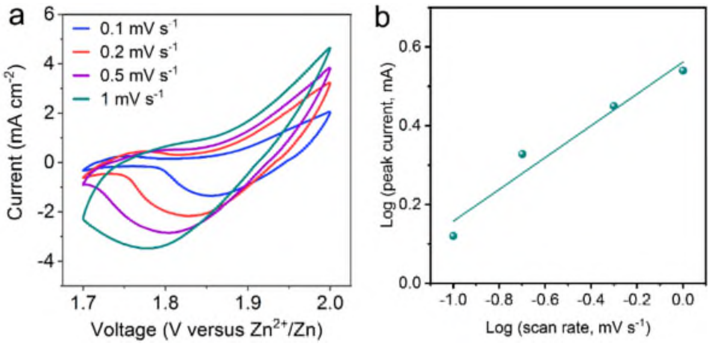

Supplementary Figure 6. (a) CV profiles of the Cl-redox reaction at different scanning rates. (b) The b-value is calculated based on the CV profiles in (a) as the value of 0.41, featuring the conversion reactions with diffusion determined behavior. Specifically, the charge storage kinetics at these three cathodic peaks (*I*) according to the current dependence on the scan rate (*v*) as $I = a\ v^b$, where *a* is a coefficient and the *b* value of 0.5 indicates semi-infinite diffusion determined behavior, while b of 1 implies capacitive behavior. It was tested in two-electrode pouch Zn||Cl-I cells cycled in 30 m $ZnCl_2$ at 0.5 A g_{I2}^{-1} and 25 °C.

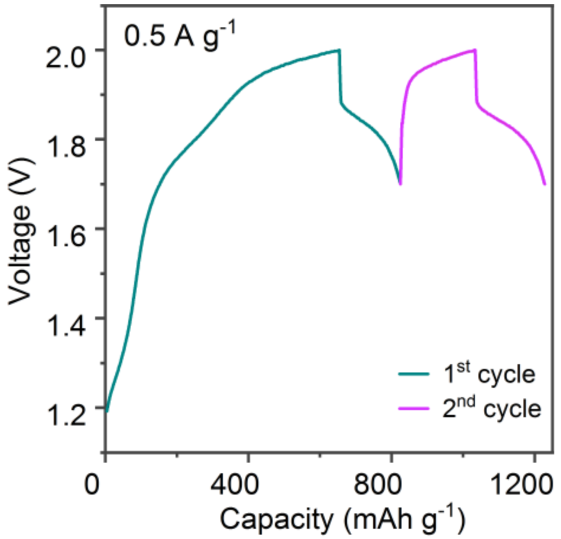

Supplementary Figure 7. The GCD curves of the first and second cycles, while the charging process started from the open circuit potential at 1.15 V and discharging process ends at 1.7 V, explaining the low coulombic efficiency of the first cycle. Of note, the Coulombic efficiency can be enhanced by regulating the discharging ending voltage to 0.6 V, as evidenced in Fig. 5 in main text. It was tested in two-electrode pouch Zn||Cl-I cells cycled in 30 m $ZnCl_2$ at 0.5 A g_{I2}^{-1} and 25 °C, where the specific current and specific capacity was based on the mass of iodine.

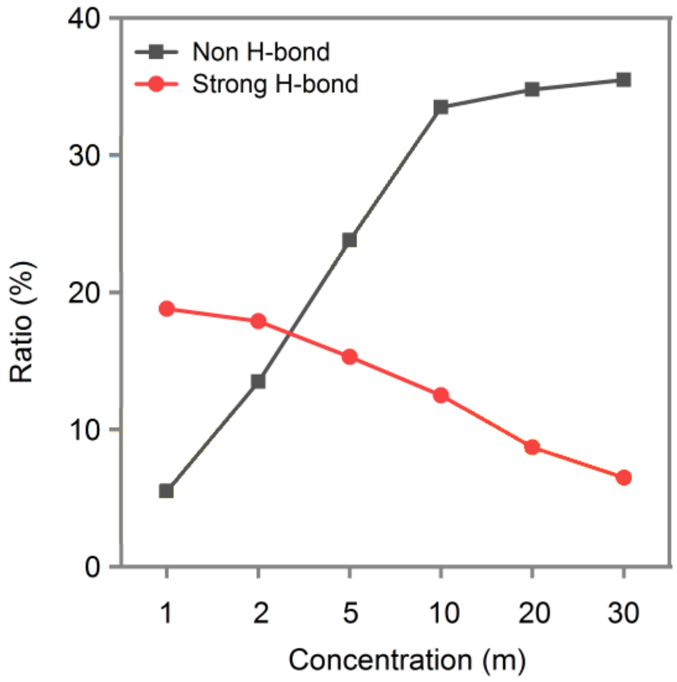

Supplementary Figure 8. The variation of strong-bonds and non-hydrogen bonds along increasing the electrolyte concentrations from 1 m to 30 m. Regarding the Raman results presented in Figure 2d in maintext, there are three kinds of H_2O molecular interactions in the aqueous electrolyte, as the strong hydrogen bonds (centered at 3325 cm^{-1}), the weak hydrogen bonds (centered at 3429 cm^{-1}) and the non-hydrogen bonds (centered at 3593 cm^{-1}). A low ratio of strong-H bonds corresponded to low water activity and consequently low dissolving capability for the halogen-based species.

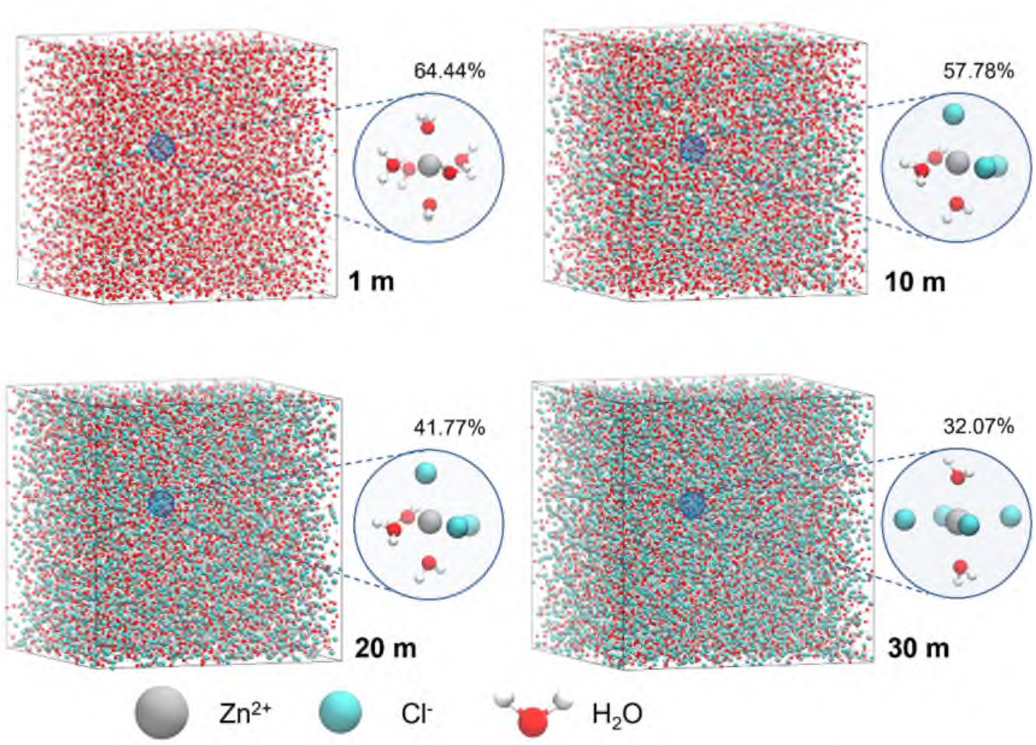

Supplementary Figure 9. Snapshots of the simulation box for systems in different concentrations as 1 m, 10 m, 20 m, and 30 m were $ZnCl_2$ electrolytes, and the zoom-in images are the corresponding major solvation structures of Zn^{2+} and their proportions of the electrolyte system.

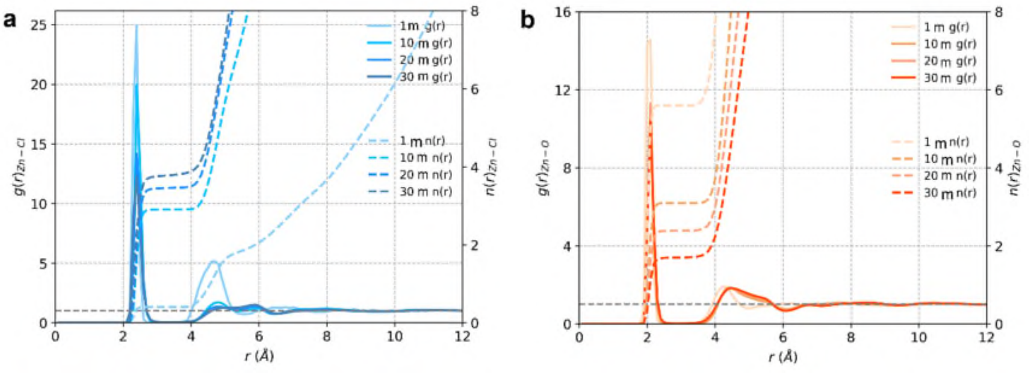

Supplementary Figure 10. Radial distribution functions (RDFs, g(r)) and coordination number (n(r)) of Zn-Cl (a) and Zn-O (H_2O) (b).

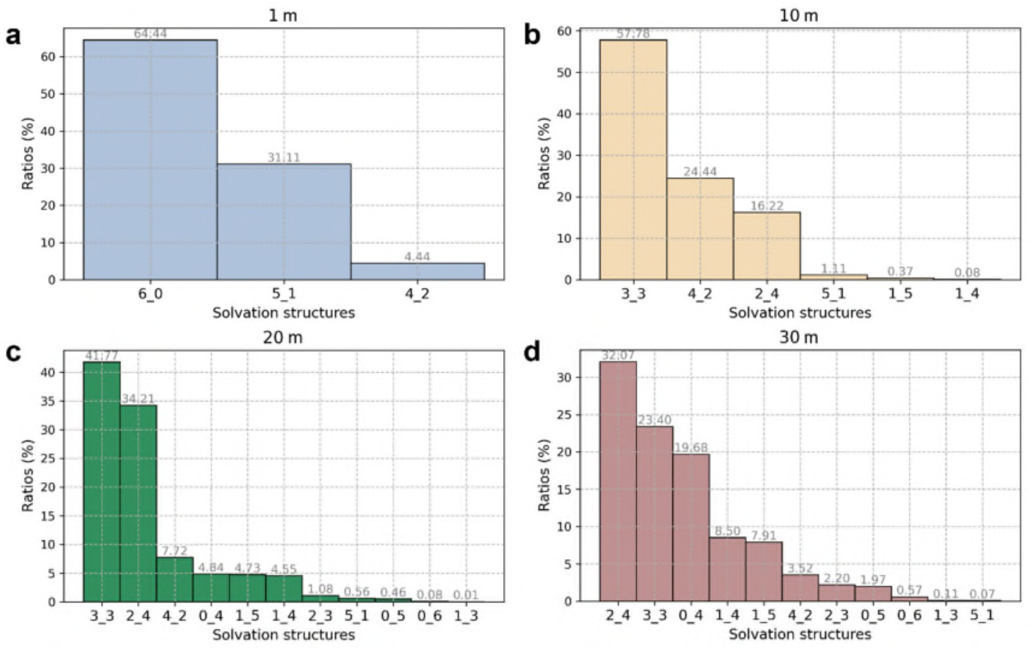

Supplementary Figure 11. The ratio distribution of different solvation structures in systems of different concentrations. The number combination “m_n” corresponds to the solvation structure $[Zn(H_2O)_mCl_n]^{(2-n)}$, For example, “2_4” stands for $[Zn(H_2O)_2Cl_4]^{2-}$.

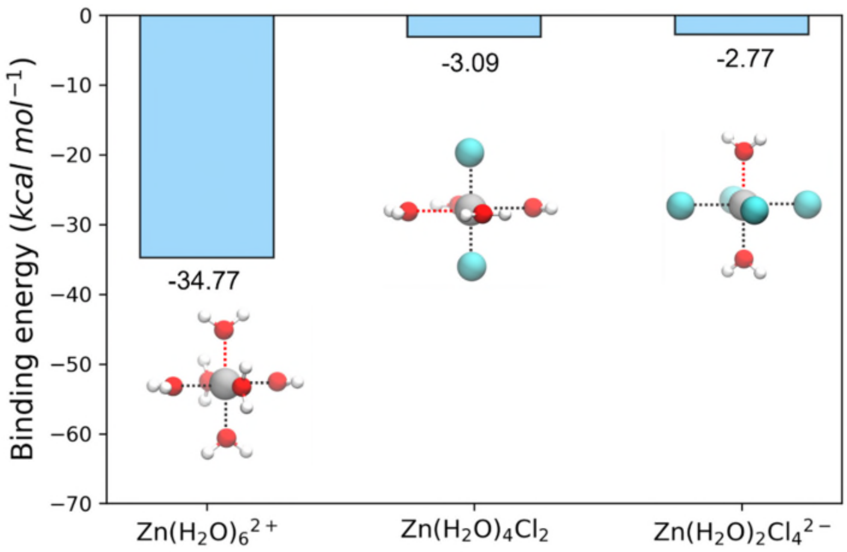

Supplementary Figure 12. The binding energy of one water molecule with the different solvation structures.

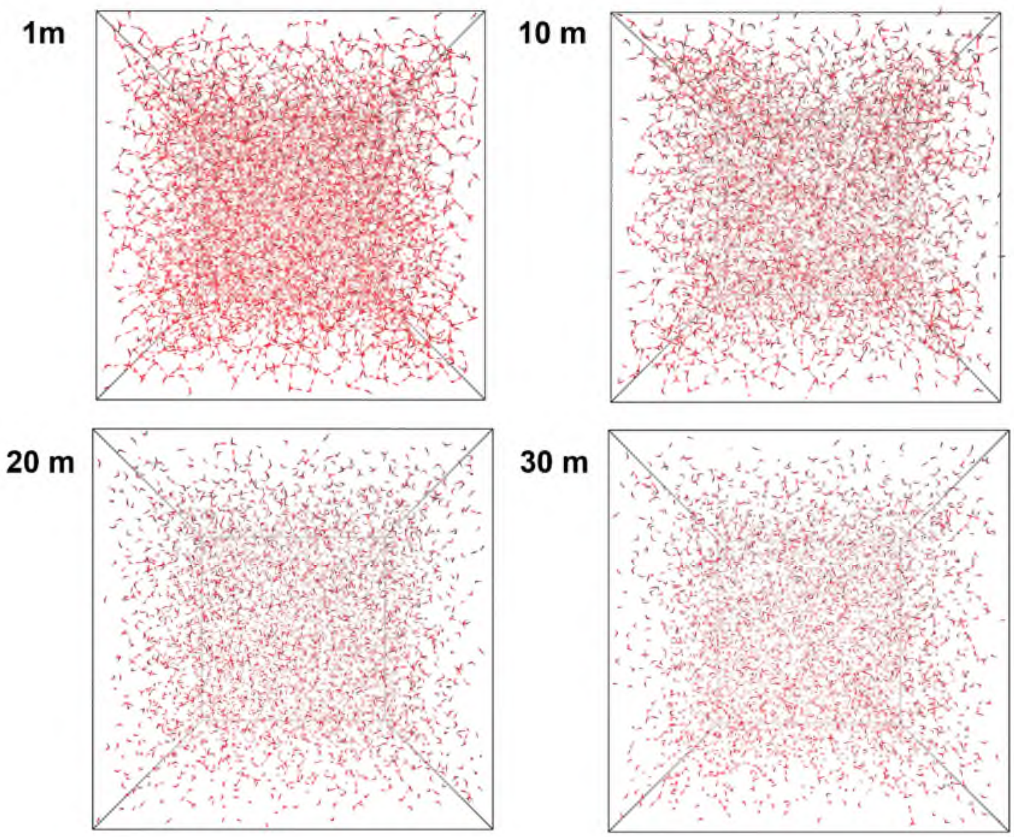

Supplementary Figure 13. Snapshots of hydrogen bonds in simulation boxes of different electrolyte systems.

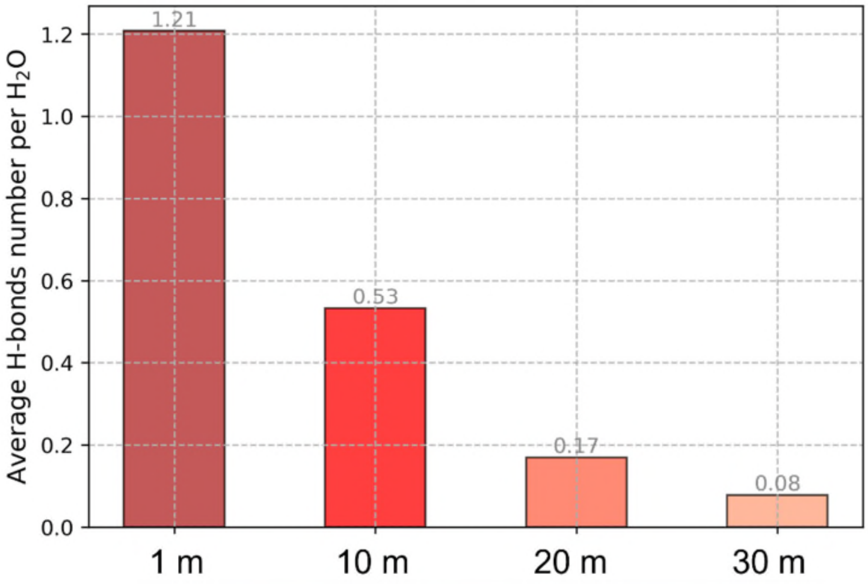

Supplementary Figure 14. Average H-bonds number per H_2O in systems of different electrolyte concentrations.

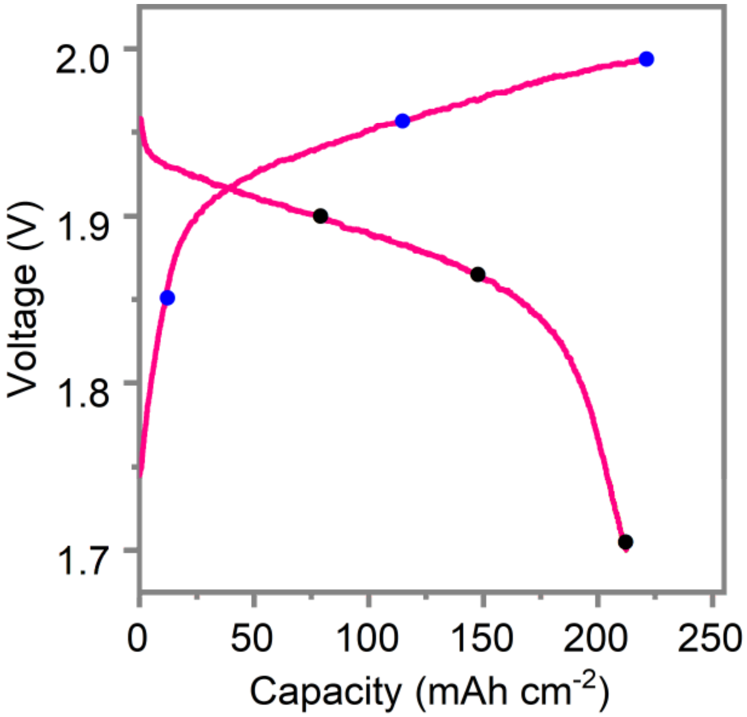

Supplementary Figure 15. The GCD curves corresponded to the voltage points of different in situ and ex situ characterizations with the operating specific current at 0.5 A g^{-1}. The voltage values captured during charging cycle are marked out by blue points as 1.85 V, 1.95 V and 2 V, while they are the black points for discharging cycle as 1.9 V, 1.85 V and 1.7 V, respectively. It was tested in two-electrode pouch Zn||Cl-I cells cycled in 30 m $ZnCl_2$ at 0.5 A g_{I2}^{-1} and 25 °C, where the specific current and specific capacity was based on the mass of iodine.

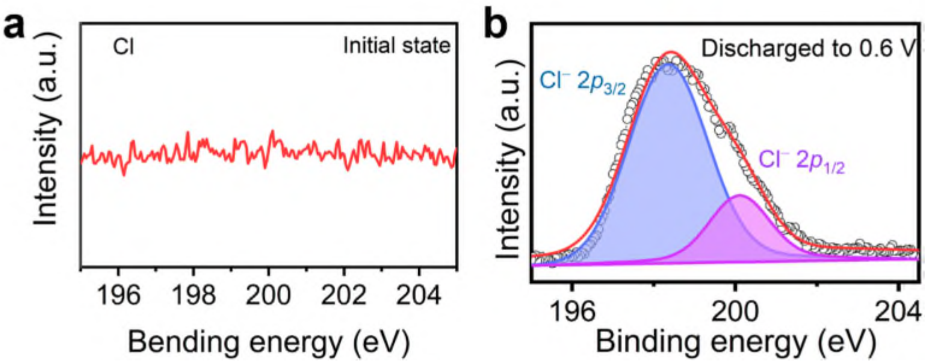

Supplementary Figure 16. The ex situ XPS results of the initial state and discharged state of Cl elements. (a) No Cl element was detected at the initial state because the electrode was prepared by electrodepositing I_2 in ZnI_2 solution. (b) There was only Cl- ion detected at fully discharged state (0.6 V) without the Cl^0 detected. It was tested in two-electrode pouch Zn||Cl-I cells cycled in 30 m $ZnCl_2$ at 0.5 A g_{I2}^{-1} and 25 °C, which was cycled for 20 cycles before the cell disassembly.

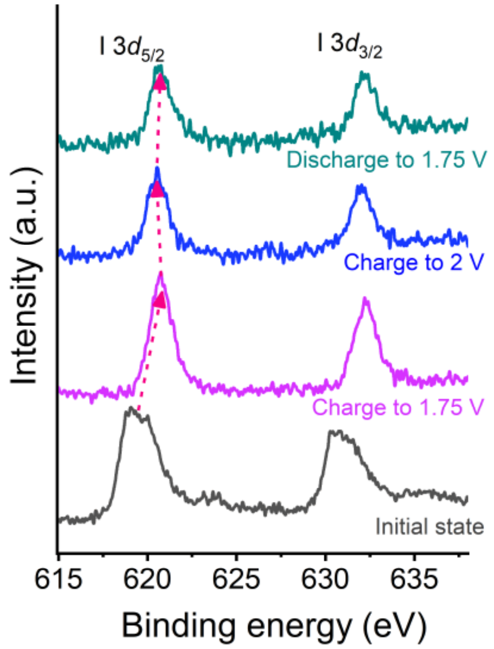

Supplementary Figure 17. The ex situ XPS results of iodine elements in different charged-discharged states. The peak of I 3$d_{5/2}$ shifted to higher binding energy (BE) with the electrode charged from initial state to 1.75 V, and then shifted to lower values when further charged to 2 V. It reversed back to higher BE after discharged back to 1.75 V. The whole process is arrowed out. It was tested in two-electrode pouch Zn||Cl-I cells cycled in 30 m $ZnCl_2$ at 0.5 A g_{I2}^{-1} and 25 °C, which was cycled for 20 cycles before the cell disassembly.

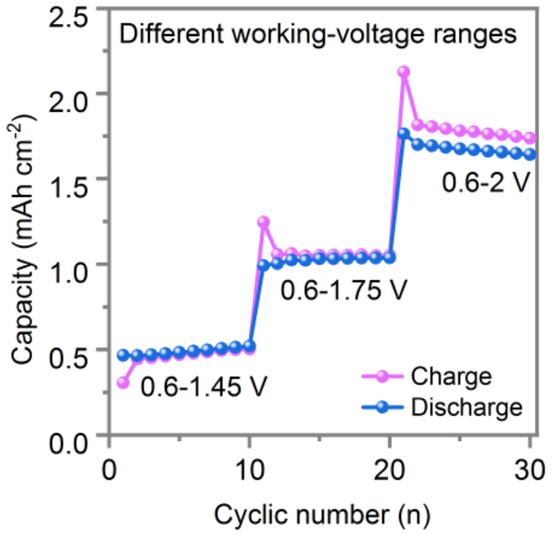

Supplementary Figure 18. Cycling performance of different working ranges to include 1, 2, 3-electrons reaction, respectively, in one cell. It was tested in two-electrode pouch Zn||Cl-I cells cycled in 30 m $ZnCl_2$ at 0.5 A g_{I2}^{-1} and 25 °C.

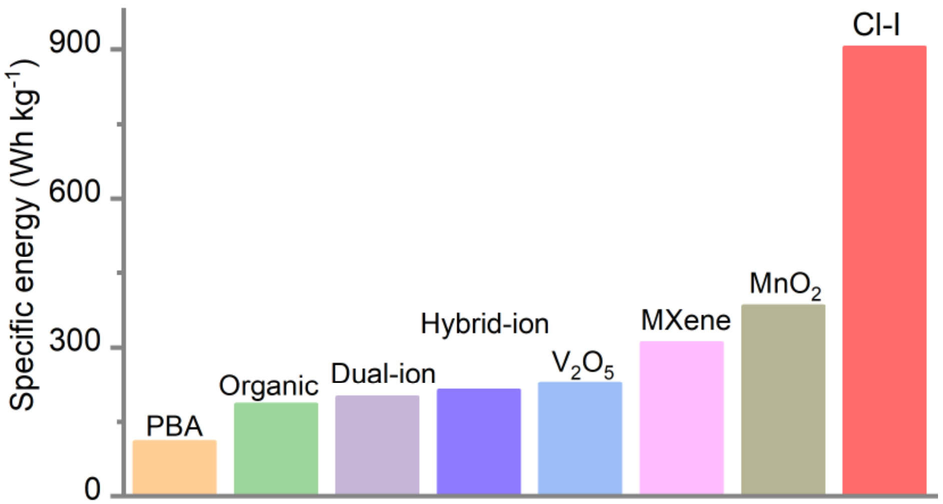

Supplementary Figure 19. Performance comparison between Cl-I cathode with other advanced cathode materials in aqueous Zn-based battery systems, where the specific values of specific energy are based on the mass of the active material in the positive electrode, as provided in the following Supplementary Table 3.

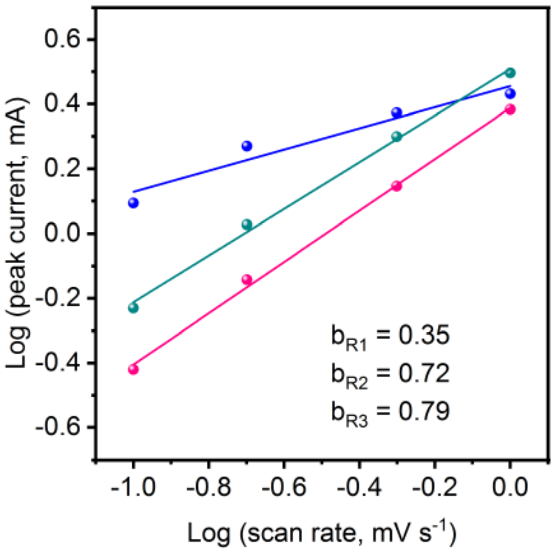

Supplementary Figure 20. The *b*-values of three cathodic peaks were correlated to the CV profiles in Figure 5a.

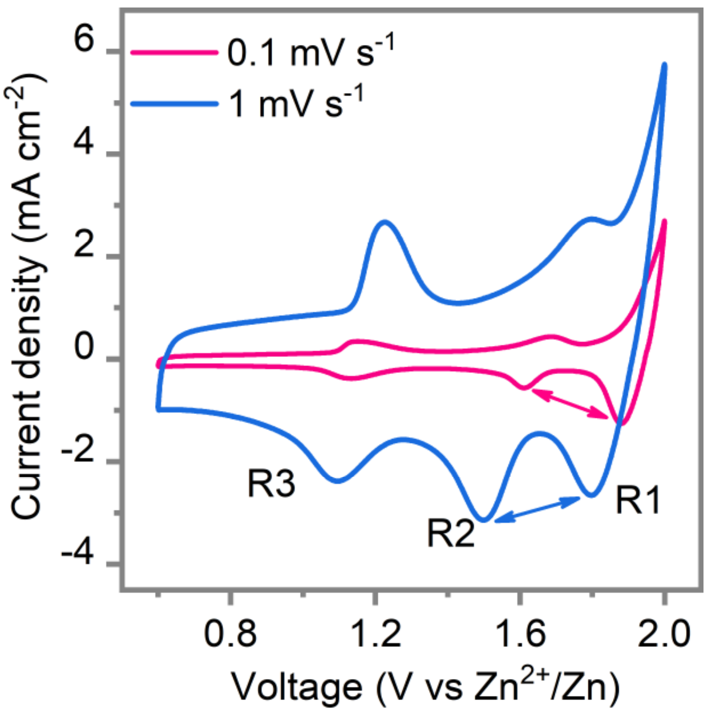

Supplementary Figure 21. Close observations of CV curves at 0.1 mV s^{-1} and 1 mV s^{-1}. The arrows pointed out the changing tendency at different scanning rates of the current intensities of the peak R1 and R2. It was tested in two-electrode pouch Zn||Cl-I cells cycled in 30 m $ZnCl_2$ at 0.5 A g_{I2}^{-1} and 25 °C.

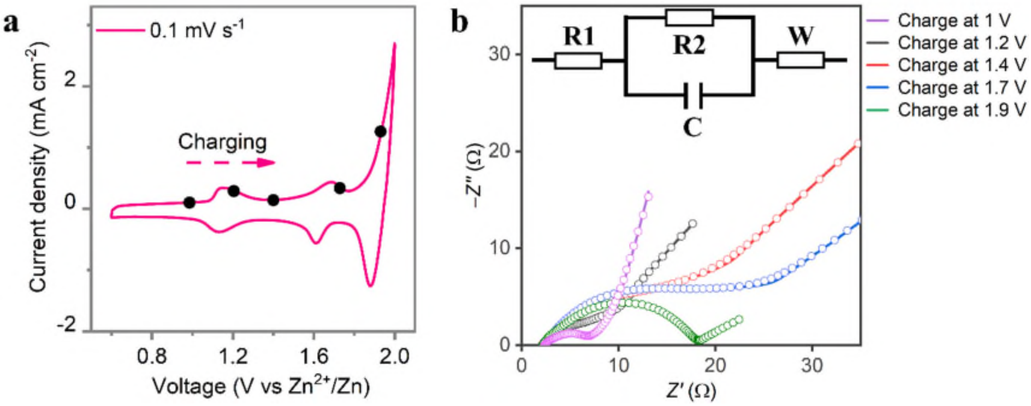

Supplementary Figure 22. The charge transfer resistance of the charge process. (a) The CV curve of Cl-I electrode. (b) Nyquist plots at different charge states pointed out at the CV curve with raw impedance data reported as symbols and fitted data as lines. The inset is the equivalent circuit element fitting the EIS data, where R1 is the ohmic resistance, R2 is the charge transfer resistance, C is the capacitor and W is the Warburg element. The ionic transfer resistance, *i.e.*, the R2 increased during charging. The fitted resistance values of R1 and R2 are presented in Supplementary Table 4.

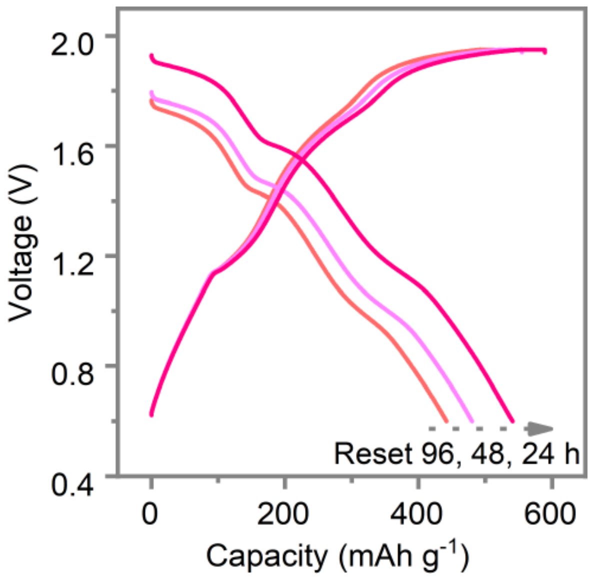

Supplementary Figure 23. The GCD curves of full cell after discharging for different hours. It was tested in two-electrode pouch Zn||Cl-I cells cycled in 30 m $ZnCl_2$ at 0.5 A g_{I2}^{-1} and 25 °C, where the specific current and specific capacity was based on the mass of iodine.

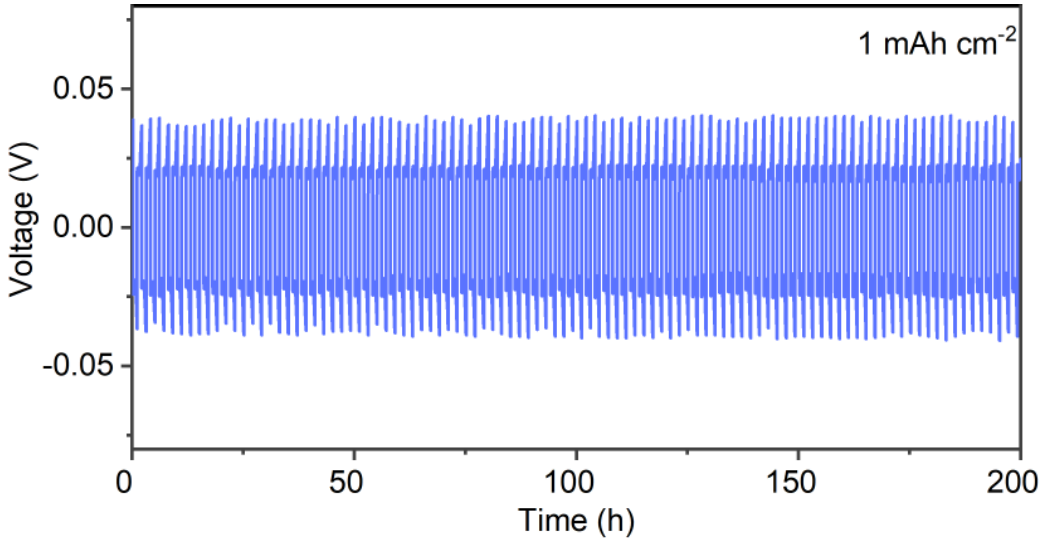

Supplementary Figure 24. The cycling stability of Zn||Zn symmetric cell in coin cells cycled in 30 m $ZnCl_2$ at the current density of 1 mA cm^{-2} and the areal capacity of 1 mAh cm^{-2} and 25 °C.

Supplementary Note 1. When the capacity calculation involves the anions from the electrolyte, it is a general protocol not to count the ion masses resourcing from the electrolyte. For a representative example, the cathode capacity to host the $AlCl_4^-$ anions in the aluminum ion battery was calculated based on the mass of the host active materials, such as graphite[13,14], and the TiS_x [15], without counting the $AlCl_4^-$ ions resourcing from the electrolyte. In addition, regarding the other anions-based batteries, the specific capacity was also calculated based on the masses of the graphite cathode without counting the masses of the anions from the electrolyte[16].

We have specified that the specific capacity is calculated based on the mass of fixing agents of iodine. The mass of Cl^- was not counted in the total mass to calculate the specific capacity, because the Cl^- ions are resourced from the electrolyte, moving into the cathode side during the charging process and returning to the electrolyte during discharging. The mass of chloride ions involving the electrode reaction is always changing during the charge-discharge process and therefore, the total mass of Cl changes during battery operation, while the mass of I is fixed. Thus, we only used the mass of I, which is regarded as a constant mass value.

Supplementary Note 2. The potential shift tendency of the oxidation reaction of Cl^0/Cl^- can be explained by the Nernst equation, while the reaction is expressed as below:

$Cl^0 + e^- \rightleftharpoons Cl^-$

The Nernst Equation is:

$$\varphi = \varphi^\theta + \frac{2.303RT}{nF} lg \frac{[Cl0]}{[Cl-]} = \varphi^\theta + \frac{0.0592}{n} lg \frac{[Cl0]}{[Cl-]}$$

Where φ is electrode reaction potential; φ^θ is the standard potential; R is the universe gas constant as 8.314 J K^{-1} mol^{-1}; T is the temperature: 298.15 K; F is the Faraday constant: 96500 C mol^{-1}. The activity of fixed Cl^0 is considered as 1, thus the equation can be further simplified as: $\varphi = \varphi^\theta - 0.0592\ lg[Cl^-]$

Therefore, the reaction potential of Cl^0/Cl^- conversion reaction would shift to lower potential by increasing the concentration of Cl^- ion.

Supplementary Note 3. The binding energy of one water molecule with the rest structure of

the corresponding solvation shell is calculated using the following equation:

$$E_b = E_{sol} - E_{sol*} - E_{H_2O}$$

where E_{sol} is the single point energy of the optimized solvation structure, E_{sol*} represents the single point energy of the structure with one water molecule excluded from the optimized solvation structure, and E_{H_2O} represents the single point energy of the water molecule in the solvation structure.

Supplementary Note 4. Regarding the XPS results of one specific element, the binding energy (BE) of higher value corresponds to higher valence state throughout the oxidation process. On the contrary, when the valence state becomes lower, BE values will become smaller. The BE variations of I is in line with this characteristic. In specific, when the initial I_2 is oxidized to 1.75 V to produce I^+, the BE becomes larger; when the oxidation continues to 2 V, the BE becomes smaller, which can be assigned to the decrement in valence state of I. The XPS results is in consistence with the charge distributions of final products as ICl_3^- at the charged state of 2 V, where the valence state of I becomes smaller from +1 at 1.75 V to +0.79 at 2 V (as exhibited in Fig 3e)

Supplementary Note 5. It is confirmed the ultimately stable products as ICl_3^- throughout the halogen-based redox reaction, which is the negatively charged anions. In order to keep electrical neutrality, they are possible to coordinate the cationic Zn^{2+}-ions in the electrolyte, and the possible final form could be $Zn(ICl_3)_2$.

Thus, the full-cell reaction equations of the Cl/I-Zn ion battery is

$$2I^- + 6Cl^- + 4Zn^{2+} \rightleftharpoons Zn(ICl_3)_2 + 3Zn$$

Supplementary Note 6.

Capacity Calculation. The specific capacity and specific energy were calculated based on the mass of iodine, while the mass of activated carbon (AC) was not counted in. Since the mass of electrodeposited iodine is 2.36 mg cm^{-2} and the mass of AC is 4 mg cm^{-2}, the specific capacity of the Cl-I electrode based on the total mass of iodine and AC at a specific current would be

$$C_T = C_I * 2.36/(2.36+4) = 0.37C_I$$

where the C_I is the capacity calculated based on the mass of iodine and C_T is the capacity calculated total mass based on iodine and AC. Thus, the corresponding capacity based on the total mass of iodine and AC is 226 mAh $g_{(I+AC)}^{-1}$ at 0.5 A g^{-1}, and the corresponding capacities at other specific currents are shown in Supplementary Table 5.

Specific Energy Calculation.. The specific energy was calculated based on the mass of iodine, where the mass of activated carbon (AC) and the mass of Zn anode were not counted in. Since the mass of electrodeposited iodine is 2.36 mg cm^{-2} and the mass of AC is 4 mg cm^{-2} and the mass of zinc foil is 35.9 mg cm^{-2} (50 μm thickness), the specific energy of the full cell based on the total mass of cathode (mass of iodine and AC) and anode materials would be

$$E_T = E_I * 2.36/(2.36+4+35.9) = 0.056E_I$$

where the E_I is the specific energy calculated based on the mass of iodine and E_T is the specific energy calculated based on the total mass. Thus, the specific energy of the Zn||I_2 cells based on the positive electrode (mass of iodine and AC) and negative electrode active materials is 50.7 Wh kg^{-1}.

In order to improve the specific energy of the whole battery, promising strategies can mainly be improved from two aspects, one is to increase the loading of the halogen species or to reduce the mass of hosting materials, and the second is to increase the utilization rate of the Zn anode.

Supplementary Note 7. The pouch cell was fabricated to demonstrate the scalability of the Cl-I electrode obtained by electrochemical deposition, which could be cycled in the size of 80 cm^2 (10 cm * 8 cm). Other applicable methods to deposit iodine into the host carbon can also be developed, such as thermal-evaporation deposition or immersion deposition method.

Supplementary Table 1. Reaction Formula of Different Electron Reaction.

Redox species	Reaction Formula
$I^- \leftrightarrow I^0$ (1st electron)	$I^- \rightleftharpoons I^0 + e^-$ (oxidation step)
$I^0 \leftrightarrow I^+$ (2nd electron)	$I^0 + Cl^- \rightleftharpoons I^+Cl^- + e^-$ (oxidation step)<br>$I^+Cl^- + Cl^- \rightleftharpoons ICl_2^-$ (stabilization step)
$Cl^- \leftrightarrow Cl^0$ (3rd electron)	$I^0 + Cl^- \rightleftharpoons I^+Cl^- + e^-$ (oxidation step)<br>$I^+Cl^- + Cl^- \rightleftharpoons ICl_2^-$ (stabilization step)

Supplementary Table 2. Comparison between different halogen-based batteries.

Parameters / Works	Redox species	Transferred electron numbers (based on redox center)	Specific energy ((based on practical value)	Applied electrolyte	Battery systems
Reference 17	Iodine	Two	750 Wh kg^{-1} (based on I mass)	Bisalt ($ZnCl_2$ and LiCl) in CH_3CN	Organic I_2‖Zn battery
Reference 18	Chlorine	One	1700 Wh kg^{-1} (based on carbon mass)	Bisalt (NaFSI and NaTFSI) in $SOCl_2$	Organic Cl_2‖Na battery
Our work	Iodine & Chlorine	Three (2 from I and 1 from Cl)	905 Wh kg^{-1} (based on I mass)	30 M $ZnCl_2$ in H_2O	Aqueous $(ClI_3)^{2-}$‖Zn battery

Supplementary Table 3. Representative and Advanced Cathode Materials in Aqueous Zn-based Battery Systems.

Parameters / Positive electrode	Capacity (mAh g^{-1})	Average voltage (V)	Specific energy (Wh kg^{-1}) (Practical value)	Applied electrolyte and negative electrode	Reference
Prussian blue Analogues (PBA) as iron hexacyanoferrate	74.6	1.45	108.2	Zinc; 20 M LiTFSI + 1 M $Zn(TFSI)_2$ in H_2O	Ref. 19 in Adv. Mater. in 2019
Organic electrode as $Cu_3(HHTP)_2$	228	0.75	171	Zn; 3 M $Zn(OTF)_2$ in H_2O	Ref. 20 in Nat. Commu. in 2019
Dual ion host electrode as graphite	110	2	200	Zn; 20 m NaFSI + 0.5 m $Zn(TFSI)_2$ in H_2O	Ref. 21 in Adv. Energy. Mater. in 2020
Hybrid ion electrode as $LiMn_2O_4$	118.8	1.7	214	Zn; 20 m LiTFSI + 1 m $Zn(TFSI)_2$ in H_2O	Ref. 22 in Nat. Mater. in 2018
V_2O_5	228	0.81	228	Zn; 1 M $ZnSO_4$ in H_2O	Ref. 23 in Nat. Energy in 2016
MXene as V_2CT_x	410	0.75	310.3	Zn; 20 m LiTFSI + 1 m $Zn(TFSI)_2$ in H_2O	Ref. 24 in Adv. Energy. Mater. in 2020
MnO_2	285	1.35	384	Zn; 2 M $ZnSO_4$ + 0.1 M $MnSO_4$ in H_2O	Ref. 25 in Nat. Energy in 2016
Cl-I	612.5	1.48	905	Zn; 30 m $ZnCl_2$	***This work***

Supplementary Table 4. Comparison of resistance values of the Zn||Cl-I pouch cell at the different charged states, which were corresponded to the EIS results in Supplementary Figure 22.

Charged state	Intrinsic resistance (R1, Ω)	Charge transfer resistance (R1, Ω)
1 V	2.66	5.3
1.2 V	2.67	8.2
1.4 V	2.68	16.5
1.7 V	2.69	21.2
1.9 V	2.69	18.3

Supplementary Table 5. Performance of advanced cathode materials in aqueous battery systems.

Parameters / Battery negative ‖ positive	Capacity (mAh g^{-1})	Average voltage (V)	Specific energy (Wh kg^{-1}) (Practical value)	Applied electrolyte	Reference
LiBr-LiCl ‖ Graphite	243	4.1	996	21 m LiTFST + 7 m LiOTF in H_2O	26
$LiMn_2O_4$ ‖ $Li_4Ti_5O_4$	88	2.3	202	2 m LiTFSI in 94% PEG + 6% H_2O	27
$LiMn_2O_4$ ‖ c-TiO_2	95	2.1	199.5	32 m KOAc+ 8 m LiOAc in H_2O	28
$LiMn_2O_4$ ‖ Zn	118.8	1.7	214	20 m LiTFSI + 1 m $Zn(TFSI)_2$ in H_2O	22
NaMnPBA‖$NaTiOPO_4$	140	1.74	199.5	9 m NaOTF + 22 m TEAOTF in H_2O	29
KFeMnPBA‖PTCDI	120	1.27	152	22 M KOTF in H_2O	30
FePBA ‖ Zn	76	1.45	110	20 M LiTFSI + 1 M $Zn(TFSI)_2$ in H_2O	19
$Cu_3(HHTP)_2$ ‖ Zn	228	0.75	211	3 M $Zn(OTF)_2$ in H_2O	20
MnO_2 ‖ Zn	285	1.35	384	2 M $ZnSO_4$ + 0.1 M $MnSO_4$ in H_2O	25
$Zn_{0.25}V_2O_5$ ‖ Zn	282	0.81	228.5	1 M $ZnSO_4$ in H_2O	23
NaV_3O_8 ‖ Zn	380	0.8	304	1 M $ZnSO_4$ + 1 M Na_2SO_4 in H_2O	31
VOOH ‖ Zn	426	0.75	336	3 M $Zn(OTF)_2$ in H_2O	32

Cl-I ‖ Zn	612.5	1.48	905	30 m $ZnCl_2$ in H_2O	***This work***

Supplementary Table 6. The specific capacities based on the iodine mass and the total mass of iodine and activated carbon.

Specific Capacity / Specific Currents* (A g^{-1})	Based on iodine mass (mAh g^{-1})	Based on total mass of iodine and Activated carbon# (mAh g^{-1})
0.5	612	226
1	573	212
2	525	194
4	482	178
8	455	168

*The specific current values refer to the mass of iodine in the positive electrode

#Since the mass of electrodeposited iodine is 2.36 mg cm^{-2} and the mass of AC is 4 mg cm^{-2}, the total output capacity is not changed, the specific capacity of the Cl-I electrode at specific current density would be

$$C_T=C_I*2.36/(2.36+4)=0.37C_I$$

where the C_I is the capacity calculated based on the mass of iodine and C_T is the capacity calculated total mass based on iodine and AC.

Supplementary Table 7. Numbers of ions and water molecules in the simulation systems of different concentrations.

Concentration	Zn^{2+}	Cl^{-}	water
1 m	90	180	5000
10 m	900	1800	5000
20 m	1800	3600	5000
30 m	2700	5400	5000

Supplementary Reference List

1. Plton S., Fast parallel algorithms for short-range molecular dynamics. *Journal of Computational Physic,* **117,** 1-19 (1995).
2. Li, P., Roberts B.P., Chakravorty, D.K., Merz, & Rational, Jr. Design of Particle Mesh Ewald Compatible Lennard-Jones Parameters for +2 Metal Cations in Explicit Solvent. *J. Chem. Theory Comput.* **9,** 2733-2748 (2013).
3. Li, P., Song, L.F., & Merz, Jr. Systematic Parameterization of Monovalent Ions Employing the Nonbonded Model. *J. Chem. Theory Comput.* **11,** 1645-1657 (2015).
4. Smith, P., Ziolek, R.M., Gazzarrini, E., Owen, D.M. & Lorenz, C.D. On the interaction of hyaluronic acid with synovial fluid lipid membranes. *Phys. Chem. Chem. Phys.* **21,** 9845-9857 (2019).
5. Michaud-Agrawal, N., Denning, E.J., Woolf, T.B., & Beckstein O. MD Analysis: a toolkit for the analysis of molecular dynamics simulations. *J. Comput. Chem.* **32,** 2319-2327 (2011).
6. Frisch, M.J. *et al.* Gaussian 09 Rev. D.01, in, Wallingford, CT, 2009.
7. Zhang, Q., *et al.* Modulating electrolyte structure for ultralow temperature aqueous zinc batteries. *Nat. Commun.* **11** 4463 (2020).
8. Zhang, Y., He, H., Dong, K., Fan, M. & Zhang, S. A DFT study on lignin dissolution in imidazolium-based ionic liquids. *RSC Adv.* **7** 12670-12681 (2017).
9. Humphrey, W., Dalke, A. & Schulten, K. VMD: Visual molecular dynamics, *J. Mol. Graph. Model.* **14,** 33-38 (1996).
10. Frisch, M. et al. J. Gaussian 09, Revision B.01, Gaussian, Inc., Wallingford CT, 2010.
11. Lee, C., Yang, W. & Parr, R. G. Development of the Colle-Salvetti correlation-energy formula into a functional of the electron density. *Physical Review B* **37**, 785-789 (1988).
12. Becke, A. D. A new mixing of Hartree–Fock and local density-functional theories. *J. Chem. Phys.* **98**, 1372-1377 (1993).
13. Lin, MC., *et al.* An ultrafast rechargeable aluminium-ion battery. *Nature* **520**, 324–328 (2015).
14. Wang, DY., *et al.* Advanced rechargeable aluminium ion battery with a high-quality natural graphite cathode. *Nat. Commun.* **8**, 14283 (2017).
15. Sun X., *et al*. Tailoring electronic-ionic local environment for solid-state Li-O_2 battery by engineering crystal structure, *Sci. Adv.* **8,** 35 (2022)
16. Huang, Z., *et al.* Manipulating anion intercalation enables a high-voltage aqueous dual ion battery. *Nat. Commun.* **12,** 3106 (2021).
17. Zou, Y., *et al.* A four-electron Zn-I_2 aqueous battery enabled by reversible $I^-/I_2/I^+$ conversion. *Nat. Commun.* **12,** 170 (2021).
18. Zhu, G., *et al.* Rechargeable Na/Cl_2 and Li/Cl_2 batteries. *Nature* **596**, 525–530 (2021).
19. Yang, Q., *et al.* Activating C-Coordinated Iron of Iron Hexacyanoferrate for Zn Hybrid-Ion Batteries with 10 000-Cycle Lifespan and Superior Rate Capability. *Adv. Mater.* **31**, 1901521 (2019).
20. Nam, K. W., *et al.* Conductive 2D metal-organic framework for high-performance cathodes in aqueous rechargeable zinc batteries. *Nat. Commun.* 2019, **10**, 4948 (2019).

21. Rodríguez-Pérez, I. A. *et al.* Enabling Natural Graphite in High-Voltage Aqueous Graphite || Zn Metal Dual-Ion Batteries. *Adv. Energy Mater.* **10**, 2001256 (2020).

22. Wang, F., *et al.* Highly reversible zinc metal anode for aqueous batteries. *Nat.Mater.* **17**, 543-549 (2018).

23. Kundu D., B. D. Adams, V. Duffort, S. H. Vajargah & L. F. Nazar, A high-capacity and long-life aqueous rechargeable zinc battery using a metal oxide intercalation cathode. *Nat. Energy*, **1**, 16119 (2016).

24. Li X., *et al.* In Situ Electrochemical Synthesis of MXenes without Acid/Alkali Usage in/for an Aqueous Zinc Ion Battery *Adv. Energy Mater.* **10**, 2001791 (2020).

25. Pan H., *et al.* Reversible aqueous zinc/manganese oxide energy storage from conversion reactions. *Nat. Energy* **1**, 16039 (2016).

26. Yang C., *et al.* Aqueous Li-ion battery enabled by halogen conversion-intercalation chemistry in graphite. *Nature* **569**, 245-250 (2019).

27. Xie J., Liang Z. and Lu Y.-C., Molecular crowding electrolytes for high-voltage aqueous batteries. *Nat. Mater.* **19**, 1006-1011 (2020).

28. Lukatskaya M. R., *et al.* Concentrated mixed cation acetate "water-in-salt" solutions as green and low-cost high voltage electrolytes for aqueous batteries. *Energy Environ. Sci.* **11**, 2876-2883 (2018).

29. Jiang L., *et al.* High-Voltage Aqueous Na-Ion Battery Enabled by Inert-Cation-Assisted Water-in-Salt Electrolyte. *Adv. Mater.* **32**, 1904427 (2020).

30. Jiang L., *et al.* Building aqueous K-ion batteries for energy storage. *Nat. Energy* **4**, 495-503 (2019).

31. Wan F., et al. Aqueous rechargeable zinc/sodium vanadate batteries with enhanced performance from simultaneous insertion of dual carriers. *Nat. Commun.* **9**, 1656 (2018).

32. Wang L., Huang K.-W., Chen J. and Zheng J., Ultralong cycle stability of aqueous zinc-ion batteries with zinc vanadium oxide cathodes. *Sci. Adv.* **5**, eaax4279 (2019).
